# Serum IL-35 Levels Are Associated With Activity and Progression of Sarcoidosis

**DOI:** 10.3389/fimmu.2020.00977

**Published:** 2020-05-19

**Authors:** Zhao Mengmeng, Song Jiacui, Du Shanshan, Zhang Yuan, Zhou Ying, Li Qiuhong, Weng Dong, Li Hui-Ping

**Affiliations:** ^1^Department of Respiratory Medicine, Shanghai Pulmonary Hospital, School of Medicine, Tongji University, Shanghai, China; ^2^Department of Respiratory Medicine, The Third Affiliated Hospital of Soochow University, Changzhou, China; ^3^Department of Respiratory Medicine, Shanghai Pulmonary Hospital, Shanghai, China

**Keywords:** IL-35, sarcoidosis, Breg, Tfh, Treg

## Abstract

**Objective:** To investigate the relationship of interleukin (IL)-35 with sarcoidosis.

**Methods:** We enrolled 114 inpatients and outpatients with sarcoidosis at the Shanghai Pulmonary Hospital, and 24 healthy controls between March 2015 and December 2017. Serum and whole blood were collected during the follow-up period. Serum IL-35 levels were detected by ELISA. Proportions of Breg, Tfh, and Treg cells in the peripheral blood were detected using flow cytometry (FCM). The mRNA levels of p35, EBI3, and GAPDH in CD19^+^ cells and CD4^+^ cells were detected by real-time PCR. Sarcoidosis granuloma mice models were established with Propionibacterium acnes (PA) and one group was treated with IL-35 antibodies. Proportions of Breg, Tfh, and Treg cells in the peripheral blood and bronchoalveolar lavage fluid (BALF) were detected by FCM.

**Results:** The IL-35 levels and the proportions of Breg and Tfh cells in the peripheral blood of patients with active sarcoidosis were significantly higher compared to patients with stable sarcoidosis and healthy controls. Moreover, the IL-35 level in patients with progressive disease was lower than that found at the initial visit. EBI3 and p35 mRNA levels in CD19^+^ cells for patients with active sarcoidosis were significantly higher as compared to patients with stable sarcoidosis and healthy controls, while there were no significant differences in p35 and EBI3 mRNA levels in CD4^+^ cells between the three groups. In the mouse model of sarcoidosis, there were loose granulomata (macrophage accumulation in the bronchial areas and immature granuloma) after intervention with IL-35 antibodies. Meanwhile, the proportions of Breg cells in the peripheral blood and BALF of the model were significantly increased, while the proportion of Treg cells declined significantly. After intervention with IL-35 antibodies, the proportion of Breg cells in the peripheral blood of mice decreased significantly as compared to the mice not exposed to anti-IL-35 antibodies.

**Conclusion:** IL-35 levels increased significantly in the serum of patients with active sarcoidosis, and lower IL-35 levels were correlated with persistent disease. Serum IL-35 levels might be better correlated with Breg cell functions.

## Introduction

Sarcoidosis is a systemic granulomatous disease of unexplained causes, and its pathological characteristic is the presence of non-caseous epithelioid granulomas (1). Sarcoidosis can involve multiple organs of the body, which mainly affects the lung and intrathoracic lymph nodes (accounting for 90% of presenting cases). The pathogenesis of sarcoidosis is still unclear, which is currently considered to be caused by some pathogenic factors in the environment in which individuals with a particular genetic susceptibility are exposed, leading to excessive cellular immune responses, and development of sarcoidosis (2, 3).

The immunological pathogenesis of sarcoidosis is complex, involving multiple cells and cytokines. Studies in recent years have found that T helper 17 (Th17) cells and regulatory T cells (Tregs), in addition to a T helper 1 (Th1)/T helper 2 (Th2) CD4^+^ T-cell imbalance, are closely correlated with the occurrence of sarcoidosis (4).

The interleukin, IL-35 is the newest member of the IL-12 family, after its discovery by Collison in 2007 (5). IL-35 is mainly produced by Tregs, and contributes to these cells playing immunosuppressive effects, as well as limiting the differentiation and functions of Th17 cells (6). IL-35 is a heterologous dimer that is composed of Epstein-Barr virus-induced gene 3 (EBI3) and the p35 subunit (7). IL-35, together with transforming growth factor (TGF)-β and IL-10 are three important immunosuppressive cytokines (8). Tregs have immunosuppressive effects by secreting cytokines such as IL-10, IL-35, TGF-β, and fibrinogen-like protein 2 (FGL2), among others. IL-35 also contributes to the suppressive activities of Tregs (5).

Recent studies have shown that regulatory B cells (Bregs) can also secrete IL-35, and that rIL-35 (recombinant IL-35) fusion proteins can induce Breg cells to secrete IL-10 and IL-35 (9, 10). IL-35 deficiency may play important roles in certain cancers and autoimmune diseases. For example, the absence of IL-35 was associated with an increase in Th17 and Th1 cells in both uveitis and encephalitis (9, 11).

Follicular helper T cells (Tfh cells) are a group of helper T-cells that are closely associated with B cells, which influence B cell differentiation and antibody production, and mainly express the cytokine IL-21 (12, 13). Previous researchers found that Tfh cells might play an important role in the occurrence and development of autoimmune diseases (14). For instance, the number of Tfh cells increases significantly in the *in vivo* circulation of patients that present with rheumatoid arthritis, and enzyme linked immunosorbent assay (ELISA) has detected heightened levels of IL-21 (15). One study has revealed that serum amyloid P component (SAP)-deficient CD4^+^ T cells from KRN (keren, a gene of the black-bellied fruit fly Drosophila) transgenic mice do not cause symptoms of arthritis after adoptive transfer into wild-type mice. Thus, abnormalities in the number and functions of Tfh cells and its molecular markers might promote the onset and development of rheumatoid arthritis (RA) (16). However, the effects of Tfh cells in sarcoidosis remain unclear.

Sarcoidosis is associated with multiple immune cellular dysfunctions. A recent study has demonstrated that IL-35 can assist Tregs in playing an immunosuppressive role (5), as well as limiting the differentiation and functions of Th17 cells (6). However, whether IL-35 is involved in the onset of sarcoidosis, or the relationship of IL-35 with Tregs and Bregs, and whether it affects Tfh cells, which specialize in helping B cells, in the onset of sarcoidosis, are also currently unclear.

The present study set out to preliminarily investigate the effects of IL-35 at different stages of sarcoidosis and to understand its relationship with both Tregs and Bregs by detecting the levels of serum IL-35 of patients with sarcoidosis. Moreover, the effects of IL-35 were further explored by intervening in the granuloma mouse model with Propionibacterium acnes (PA) using IL-35 antibodies—an approach that might elucidate novel ideas for studying the pathogenesis of sarcoidosis.

## Materials and Methods

### Patients and Healthy Controls

All inpatients and outpatients (*n* = 114) who were diagnosed with sarcoidosis by pathological biopsy at the Shanghai Pulmonary Hospital between March 2015 and December 2017, and had been screened for serum IL-35 levels at the first visit were enrolled in this study. Serum and whole blood were collected from the study participants. Among them, patients who had been followed-up for over 6 months and re-examined for IL-35 levels after 6 months were selected to study the relationship between IL-35 levels and the prognosis of sarcoidosis. We believe that a follow-up time of <6 months is insufficient to judge the prognosis of the patient. The healthy control group consisted of 24 cases, and serum and whole blood was collected from these healthy controls. The study protocol was approved by the local Ethics Committee of the Biomedical Research Center of the Medical and Life Sciences Institute of Tongji University (2011-FK-10). All subjects signed an informed consent before participating in the study. Clinical characteristics data for patients with sarcoidosis, and healthy controls are shown in Tables 1, 2.

**Table 1:** Demographic characteristics of the patients with sarcoidosis and the healthy control group.

	**Sarcoidosis (*n* = 114)**	**Healthy control (*n* = 24)**	*P-*value^*a^
Age, year	48.70 ± 1.040	44.17 ± 2.32	0.072
Male/female	35/79	11/13	0.153
BMI (kg/m^2^)^*b^	24.39 ± 0.27	25.29 ± 0.54	0.16

*a Chi-square test was used to evaluate differences of gender distribution between two groups. T-test was used to evaluate differences of age and BMI between two groups.

*b *BMI (Body Mass Index) = weight/height^2^*.

**Table 2:** Clinical characteristics in patients with active sarcoidosis and stable sarcoidosis.

	**Active sarcoidosis (*n* = 51)**	**Stable sarcoidosis (*n* = 63)**	*P*-value^*a^
Age, year	46.86 ± 1.57	50.19 ± 1.37	0.11
Male/female	19/32	16/47	0.17
BMI (kg/m^2^)	24.15 ± 0.41	24.54 ± 0.36	0.42
Ever Smokers	10/51	18/63	0.33
CXR stage^*^^b^0/I/II/III/IV	0/6/42/3/0	0/4/49/10/0	N/A
SACE (IU/L)	88.01 ±7.84(*n* = 37)	55.34 ± 3.30(*n* = 46)	<0.0001^*c^
ESR (mm/h)	30.72 ± 3.08(*n* = 43)	14.96 ± 1.52(*n* = 48)	<0.0001^*c^
24 h urinary calcium (mmol/24 h)	5.98 ± 0.59(*n* = 41)	12.21 ± 6.63(*n* = 43)	0.36
Pulmonary involvement	51/51	62/63	
Extra Pulmonary involvement	28/51	34/63	
FVC	3.33 ± 0.21(*n* = 19)	2.89 ± 0.21(*n* = 16)	0.16
FVC%	83.70 ± 2.08(*n* = 19)	85.91 ± 4.36(*n* = 16)	0.63
FEV1	2.76 ± 0.21(*n* = 19)	2.28 ± 0.18(*n* = 16)	0.09
FEV1%	82.84 ± 2.43(*n* = 19)	82.31 ± 4.81(*n* = 16)	0.92
FEV1/FVC	82.21 ± 1.58(*n* = 19)	78.92 ± 2.44 (*n* = 16)	0.25
DLCO	22.78 ± 1.50(*n* = 17)	18.80 ± 1.08(*n* = 12)	0.057
DLCO%	94.39 ± 4.01(*n* = 17)	91.23 ± 3.43(*n* = 12)	0.57
Steroid therapy	35/51	49/63	0.27

*a Chi-square test was used to evaluate differences of gender distribution, smoker, and steroid therapy between two groups. T-test was used to evaluate other differences between two groups.

*b CXR, chest X-ray; N/A, not applicable; CXR stage: 0, no adenopathy, no lung infiltrates; stage I, hilar & mediastinal adenopathy only; stage II, hilar&mediastinal adenopathy plus lung infiltrates; stage III, lung infiltrates only; stage IV, pulmonary fibrosis.

*c *The P value is significant*.

Diagnostic criteria of sarcoidosis followed the ATS/ERS/WASOG consensus (1). Biopsies of pathological tissues showed non-caseous necrotizing granulomas, while other granulomatous diseases were excluded, including tuberculosis, fungal infections, parasitic infections, tumors and vasculitis, among others. Furthermore, sputum smear-negative active tuberculosis was excluded by quantitative TB-PCR (*Mycobacterium tuberculosis*—polymerase chain reaction) (17) and a multi-parameter scoring method, which was established in our previously published study (18).

Determination of sarcoidosis activity according to the previously published approach (1).

(I) Active stage. Symptoms of respiratory system progression or evidence of new symptoms including fever and extra-pulmonary manifestations. Imaging examinations showed that intrathoracic lesions progressed, and biological indicators, including erythrocyte sedimentation rate (ESR), serum angiotensin-converting Enzyme (SACE), serum calcium, and CD4/CD8 all increased.

(II) Stable stage. Clinical symptoms were stable, and the above biological indicators recovered to normal levels.

Determination of the prognosis of sarcoidosis. All participants were asked to follow-up regularly every 3 months, until 1 year or longer. The follow-up period of the 114 cases was 0–36 months, and the average follow-up time was 5.6 ± 0.7 months. Prognosis judgement was limited to patients who were followed-up for more than 6 months.

(I) Improved group. Chest computed tomography (CT) showed that the range of lesions shrank significantly or disappeared compared to that of the last time during the follow-up period (≥6 months), and symptoms of the patients were alleviated significantly or had disappeared.

(II) Stable group. Chest CT showed that lesions were similar compared to the lesions at the last CT and the symptoms were stable during the follow-up period (≥6 months).

(III) The group with sustained or progressed disease conditions. Two independent times showing a chest CT confirming that the lesions increased, and/or the symptoms had been continuously aggravated.

Steroid therapy for sarcoidosis followed the international consensus, including thoracic sarcoidosis patients that had pulmonary symptoms. In addition, steroid therapy was given to patients without symptoms but with persistent pulmonary infiltration or progressive pulmonary dysfunction, and for patients with cardiac disease, neurologic disease, and eye disease not responding to topical therapy, and hypercalcemia (1).

### Detection of IL-35 in the Serum of Sarcoidosis Patients

Serum was collected from patients with sarcoidosis and the control group, and then IL-35 levels were assayed using a human IL-35 specific enzyme-linked immunosorbent assay (ELISA) kit (Bluegene, China). The ELISA has a dynamic range of 30–1,000 pg/mL, a limit of detection (LOD) of 30 pg/mL, and a sensitivity of 1 pg/mL.

### Investigation of the Source and Relevant Mechanisms of IL-35 in Sarcoidosis Patients

#### Detection of the Proportions of Bregs, Tfh Cells, and Tregs in Peripheral Blood

Peripheral blood was collected from patients with sarcoidosis and the control group. The proportions of Bregs (CD19^+^CD24^+^CD38^+^), Tfh cells (CD4^+^CXCR5^+^PD1^+^), and Tregs (CD4^+^CD25^+^CD127^−^) were detected by flow cytometry (FCM). Peripheral blood mononuclear cells (PBMCs) were collected after the peripheral blood was processed by erythrocyte lysate (eBioscience, USA). The cells were re-suspended in flow cytometry staining buffer solution (eBioscience, USA), and then divided into three groups. The first group was used for labeling with anti-human CD19-PE, CD24-APC, and a CD38-FITC on the cell surface. The second group was labeled with anti-human CD4-FITC, CXCR5-PE, and PD-1-APC on the cell surface. The third group was labeled with anti-human CD4-FITC, CD127-APC, and CD25-PE on the cell surface. All antibodies for fluorescent staining were purchased from eBioscience. Fluorescence signals were detected in stained cells using a flow cytometer (Beckman Coulter, Brea, CA), and the antibody-stained cells were analyzed using the software equipped with the Beckman instrument in the flow cytometer. In the first group, B cells (CD19^+^ subsets) were gated within the lymphocyte populations in forward scatter (FSC)/side scatter (SSC) dot plot, then CD24/CD38 dot plot was drawn from the CD19^+^ gate, to gate on Bregs (CD19^+^CD24^+^CD38^+^). In the second group, CD4^+^ cells were gated within the lymphocyte populations in FSC/SSC dot plot, then CXCR5/PD-1 dot plot was drawn from the CD4^+^ gate, to gate on Tfh cells (CD4^+^CXCR5^+^PD1^+^). In the third group, CD4^+^ cells were gated within the lymphocyte populations in FSC/SSC dot plot, then CD127/CD25 dot plot was drawn from the CD4^+^ gate, to gate on Tregs (CD4^+^CD25^+^CD127^−^). The gating strategy was shown in **Figure 4**.

#### Extraction of Peripheral Blood Mononuclear Cells (PBMCs) and Immune-Magnetic Bead Sorting of CD19^+^ and CD4^+^ Cells

Sterile extraction of 5 ml of peripheral venous blood was performed from patients and healthy controls. PBMCs from patients and healthy controls were isolated using the Ficoll-Hypaque (Lymphoprep™) density gradient centrifugation procedure (STEMCELL Technologies, Vancouver, British Columbia, Canada). Some of the extracted PBMCs were used to sort CD19^+^ cells using EasySep™ Human CD19 Positive Selection Kit (EasySep, USA), while some extracted PBMCs were used to sort CD4^+^ cells using the CD4^+^ T Cell Isolation Kit, human (Miltenyi Biotech, Germany).

#### Extraction of CD19^+^ and CD4^+^ Cell RNA and Fluorescence-Based Quantitative Real-Time Polymerase Chain Reaction (PCR)

RNA of CD19^+^ and CD4^+^ cells which were sorted by magnetic beads was extracted using Trizol Reagent (Invitrogen, Germany). Reverse transcription was conducted using the ReverTra Ace qPCR RT Kit (TOYOBO, Osaka, Japan). Quantitative real-time polymerase chain reaction (qRT-PCR) was performed using Thunderbird SYBR qPCR Mix Kit (TOYOBO, Osaka, Japan) and Taqman Gene Expression Assay (Applied Biosystems). Relative gene expression levels of the IL-35 subunit (p35, EBI3) and glyceraldehyde phosphate dehydrogenase (GAPDH, housekeeping gene) in CD19^+^ and CD4^+^ cells of patients with sarcoidosis were detected by quantitative real-time PCR, and the primer sequences were shown in Table 3 (19). The PCR reaction conditions were as follows: 50 cycles of denaturation at 95°C for 60 s, annealing at 95°C for 15 s, and extension at 60°C for 60 s. Real-time PCR reaction efficiency for each target gene was estimated by the slope of the standard curve. Standard curves were designated by Ct values of cDNA serial dilutions. The relative expression levels of gene transcripts were calculated by the ΔCt and 2(−ΔCt) formulas.

**Table 3:** Forward and reverse primers of genes for real-time PCR amplification.

**Primer**		**Sequence**
GAPDH	Forward	GCAAGAGCACAAGAGGAAGA
GAPDH	Reverse	ACTGTGAGGAGGGGAGATTC
EBI3	Forward	TGGCGGCTCAGGACCTCACA
EBI3	Reverse	GGGGCTTAGGGTGGCGAGGA
P35	Forward	GCAGCCTCCTCCTTGTGG
P35	Reverse	GGGAACATTCCTGGGTCTGG

### The Role of IL-35 in Animal Models

#### Establishment of the Sarcoidosis Granuloma Mouse Model

Female SPF C57BL/6 mice aged 6–8 weeks (Shanghai SLAC Laboratory Animal Co., Ltd.) were fed and housed in different cages, and were free to drink water and eat food as needed. All animal experiments were approved by the local Experimental Animal Center of Tongji University (No. K17-016). The granuloma model was established using the method described in our previous study, Propionibacterium acnes (PA) was used to induce sarcoidosis-like granulomatous inflammation in the mouse model (20). PA (purchased from ATCC, 54 USA, batch number 6919) was cultured in Clostridium Perfringens medium at 37°C for 48 h. The bacterial suspension of PA was prepared, and then PA was inactivated at 65°C for 30 min. Three groups of wild-type C57BL/6 mice were set up that included the following: (1) PA group (*n* = 8), in which mice were pre-sensitized by intraperitoneal injection of inactivated PA (0.25 mL, 2 mg/mL) and then intratracheally inoculated with inactivated PA (0.05 mL, 10 mg/mL) on days 14, 28, and 42 after the pre-sensitization. (2) PBS (phosphate buffer solution group) (*n* = 8), in which mice received an intraperitoneal injection of PBS (0.25 mL) and an intratracheal inoculation of PBS (0.05 mL) in the same manner as the PA group. (3) PA+IL-35Ab (IL-35 antibody) group (*n* = 9), in which mice were pre-sensitized by intraperitoneal injection of inactivated PA (0.25 mL, 2 mg/mL), and then an intraperitoneal injection of anti-mouse IL-12/IL-35p35 mAb (Clone 27537; R&D Systems, 50 μg) (21) that was performed at day 14. An intratracheal inoculation of inactivated PA (0.25 mL, 2 mg/mL) was conducted after 4 h. At days 28 and 42, an intratracheal inoculation of inactivated PA (0.05 mL, 10 mg/mL) was done. At day 56, peripheral blood, alveolar lavage fluid and lung tissue specimens were collected.

#### Pathological Hematoxylin-Eosin Staining (HE Staining) of Lung Tissues

After fixing in general tissue fixative (4% paraformaldehyde, neutral pH) for 24 h, the lung tissues were embedded in paraffin, then HE staining was performed in all the mice (PA: *n* = 8, PBS: *n* = 8, PA+IL-35Ab: *n* = 9), followed by slicing and scanning using a Leica pathological slicing scanner (LEICA SCN400). According to the report by Mikawa et al., the acute lung injury (ALI) scores of the HE staining tissue sections were determined (22). All HE-stained sections were analyzed and scored for ALI severity by two experienced pathologists, who were blinded for the group allocation. ALI severity was scored based on the severity of the following four histopathological features: (I) alveolar congestion; (II) bleeding; (III) inflammatory cell infiltration; and (IV) alveolar wall thickening or a hyaline membrane formation. The specific scoring criteria for ALI in the murine experiment was shown in Supplementary File 1.

#### Bregs, Tfh, and Tregs in the Peripheral Blood and Bronchoalveolar Lavage Fluid (BALF) of Mice Were Detected by Flow Cytometry (FCM)

Bronchoalveolar lavage fluid (BALF) of mice was collected to harvest cells after centrifugation. Then the cells were divided into three groups. The first group of cells was cultured in Roswell Park Memorial Institute (RPMI) 1640 medium with cell stimulation cocktail (containing Phorbol 12-myristate 13-acetate, lonomycin, Brefeldin A, Monensin, eBioscience, USA) for 16 h. The remaining two groups of cells were cultured in RPMI 1640 medium without cell stimulation cocktail. Cultures were harvested and the cells were re-suspended in fluorescence-activated cell sorting (FACS) solution. The first group of cells was labeled by surface staining with anti-mouse B220-FITC first, and then fixed and permeabilized with IC Fixation buffer (eBioscience, USA) and permeabilization buffer (eBioscience, USA) for intracellular staining with anti-mouse IL-10-PE. The second group of cells was labeled by surface staining with anti-mouse CD4-FITC, PD-1-APC, and CXCR5-PE. The third group of cells was labeled with anti-mouse CD4-FITC and CD25-PE on the cell surface first, and then fixed and permeabilized for intranuclear staining with Foxp3-APC. All antibodies for immunofluorescent staining were purchased from eBioscience. Fluorescence signals were detected in stained cells using a flow cytometer (Beckman Coulter, Brea, CA), and the antibody-stained cells were analyzed using the software equipped with the Beckman instrument in the flow cytometer. In the first group, B220/IL-10+ dot plot was drawn from the lymphocyte populations in FSC/SSC dot plot to gate on Bregs (B220+IL-10+). In the second group, CD4^+^ cells were gated within the lymphocyte populations in FSC/SSC dot plot, then PD-1/CXCR5 dot plot was drawn from the CD4^+^ gate, to gate on Tfh cells (CD4^+^PD-1^+^CXCR5^+^). In the third group, CD4^+^ cells were gated within the lymphocyte populations in FSC/SSC dot plot, then CD25/Foxp3 dot plot was drawn from the CD4^+^ gate, to gate on Tregs (CD4^+^CD25^+^Foxp^3+^). The gating strategy was shown in **Figures 8**–**10**. Mouse peripheral blood was also collected, and PBMCs were isolated after erythrocytes were lysed. The PBMCs were analyzed by flow cytometry in a similar manner as described above.

#### Detection of IL-35 and IL-21 Levels in the BALF Supernatant of Mice by ELISA

BALF supernatant was collected from at least 5 mice in each group. IL-35 and IL-21 levels in BALF was assayed by IL-35 ELISA kit and IL-21 ELISA kit. The ELISA kit of mouse IL-35 (BioLegend, USA) has a dynamic range of 0.47–30 ng/mL and an average minimum detectable concentration of 0.08 ± 0.04 ng/mL. The ELISA kit of mouse IL-21 (Neobioscience, China) has a dynamic range of 47–6,000 pg/mL and a limit of detection (LOD) of 47 pg/mL.

### Data Analysis

Data were analyzed by Graphpad Prism8 and SPSS19.0 software. Flow cytometry data were analyzed using the software equipped with the Beckman instrument. For patients with prognosis data, comparisons of the serum IL-35 level after a follow-up for more than 6 months to serum IL-35 level at the first visit were analyzed by paired Student's *t*-test. Other two-group comparisons were analyzed using unpaired Student's *t*-test. The relationship of IL-35 with Bregs, Tfh cells, and Tregs in the peripheral blood was analyzed by Pearson's correlation analysis. Results are presented as mean ± standard error (SEM). *P*-value < 0.05 were considered significant.

## Results

### Expression of IL-35 in Patients With Sarcoidosis

**Figure 1:**
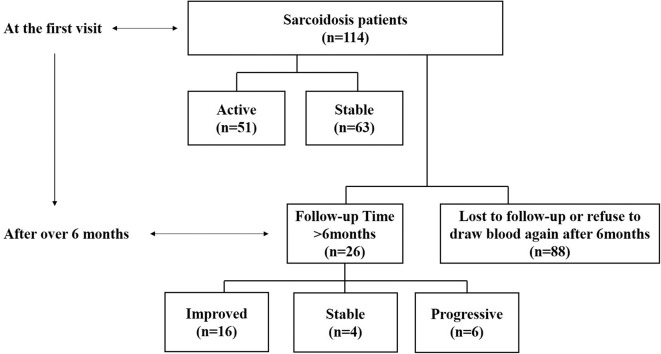
Flow diagram of the data collection and analyses. Information on 114 sarcoidosis patients and 24 healthy controls was collected. Twenty-six of the sarcoidosis patients who had been followed-up for more than 6 months and were tested IL-35 levels again after 6 months were selected to study the relationship between IL-35 levels and the prognosis of sarcoidosis patients, including 6 in the progressive group, 4 in the stable group and 16 in the improved group. The remaining 88 patients were lost to follow-up or refused to provide blood samples 6 months after the first visit.

The study included 114 sarcoidosis patients and 24 healthy controls. Among them, 26 sarcoidosis patients who had been followed-up for more than 6 months, and whose IL-35 levels were tested again after 6 months were selected to study the relationship between IL-35 levels and the prognosis of sarcoidosis, while the remaining 88 patients were lost to follow-up or refused to provide blood samples 6 months after the first visit. The patient collection flow diagram was shown in Figure 1.

#### Serum IL-35 Level Is Increased Significantly in Patients With Active Sarcoidosis

**Figure 2:**
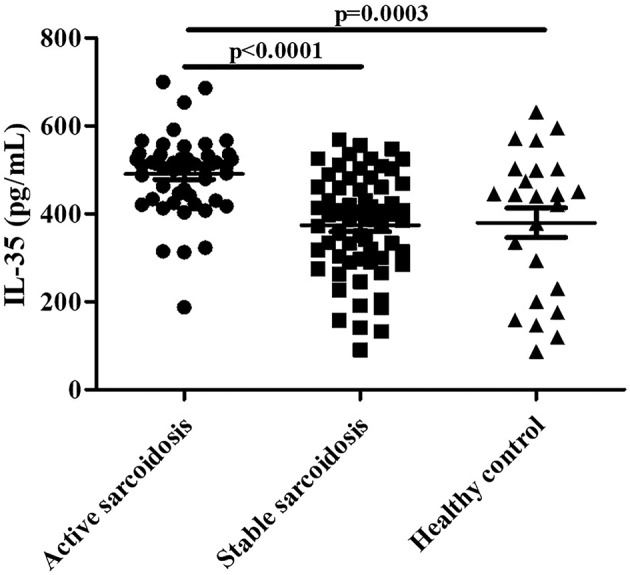
The levels of serum IL-35 in patients with active sarcoidosis (*n* = 51), stable sarcoidosis (*n* = 63), and the healthy control group (*n* = 24). The differences between two groups were analyzed by unpaired student's *t*-test.

Serum IL-35 levels in patients with active sarcoidosis were significantly higher compared to patients with stable sarcoidosis (Figure 2, *P* < 0.0001) and the healthy control group (Figure 2, *P* = 0.0003). However, there was no statistical significance between patients with stable sarcoidosis and the healthy control group.

#### Serum IL-35 Is Lower in Sarcoidosis Patients With Progressive Disease

**Figure 3:**
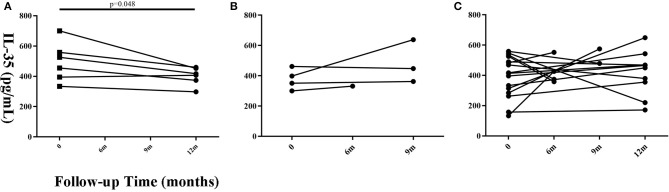
Changes in serum IL-35 concentration in patients with different prognosis at different time points during the follow-up period. **(A)** The progressive (*n* = 6), **(B)** the stable (*n* = 4), and **(C)** improved groups (*n* = 16). Time 0 denotes the first visit. The patients whose follow-up time was more than 6 months were considered suitable for prognosis judgement. Differences between the serum IL-35 levels 6 months after the first visit and the serum IL-35 levels at the first visit were analyzed by paired student's *t*-test.

Twenty-six patients with a follow-up time of ≥6 months that had prognosis data (Progressive = 6, Stable = 4, Improved = 16) were selected to observe the changing trend in serum IL-35 levels. The remaining 88 patients were lost to follow-up or refused to provide blood samples after 6 months. We believe that a follow-up time of <6 months is insufficient to judge the prognosis of the patient. The findings suggested that IL-35 level in patients with progressive disease was lower as compared to that found at the initial visit, but only 6 paired samples were available for analysis (Figure 3, paired *t*-test, *P* = 0.048). There was no significant change in serum IL-35 levels in patients with improved or stable disease.

### Investigation of the Source and Relevant Mechanisms of IL-35 in Sarcoidosis Patients

#### The Proportions of Bregs and Tfh Cells Increase Significantly in the Peripheral Blood of Patients With Active Sarcoidosis

**Figure 4:**
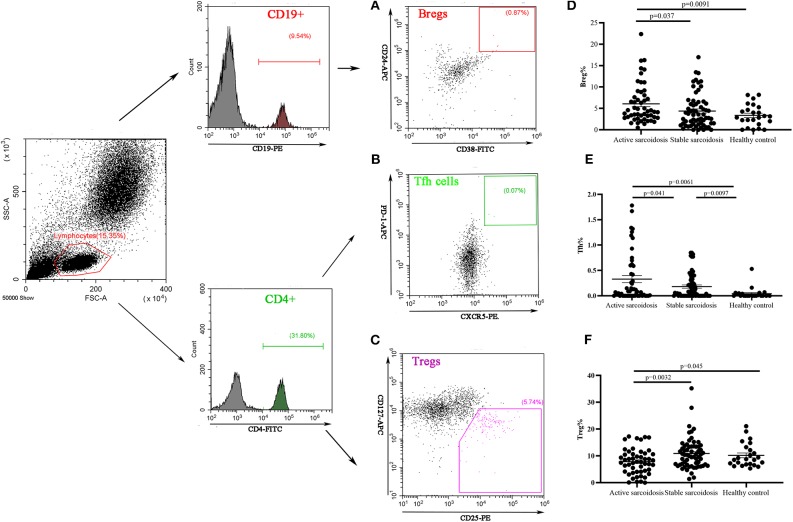
Flow cytometry analysis of Bregs, Tfh cells, and Tregs in the peripheral blood samples. **(A)** A representative comparative phenotype of CD24^+^CD38^+^ subsets produced by CD19^+^ lymphocytes from a stable sarcoidosis patient; **(B)** A representative comparative phenotype of PD-1+CXCR5+ subsets produced by CD4^+^ lymphocytes from a stable sarcoidosis patient; **(C)** A representative comparative phenotype of CD25^+^CD127− subsets produced by CD4^+^ lymphocytes from a stable sarcoidosis patient; **(D)** The proportions of Bregs in patients with active and stable sarcoidosis, and healthy controls; **(E)** The proportions of Tfh cells in patients with active and stable sarcoidosis, and healthy controls; and **(F)** The proportions of Tregs in patients with active and stable sarcoidosis, and healthy controls (active sarcoidosis: *n* = 51, stable sarcoidosis: *n* = 63, healthy controls: *n* = 24. The differences between two groups were analyzed by unpaired student's *t*-test).

The proportions of Bregs, Tfh cells, and Tregs in the peripheral blood were detected by flow cytometry (FCM). The results showed that Bregs (CD19^+^CD24^+^CD38^+^) were significantly higher in patients with active sarcoidosis as compared with patients with stable sarcoidosis (*P* = 0.037) and the healthy control group (*P* = 0.0091), while there was no significant difference when comparing stable sarcoidosis patients with the healthy control group (Figures 4A,D). Tfh cells (CD4^+^CXCR5^+^PD1^+^) in the peripheral blood of patients with active sarcoidosis were significantly higher as compared with patients with stable sarcoidosis (*P* = 0.041) and the healthy control group (*P* = 0.0061), while Tfh cells in patients with stable sarcoidosis were also higher when compared to the healthy control group (*P* = 0.0097; Figures 4B,E). Tregs (CD4^+^CD25^+^CD127^−^) in the peripheral blood of patients with active sarcoidosis were significantly lower when compared to patients with stable sarcoidosis (*P* = 0.0032) and the healthy control group (*P* = 0.045), however there was no significant difference between patients with stable sarcoidosis and the control group (Figures 4C,F).

The correlation of IL-35 levels with the proportions of Bregs, Tfh cells, and Tregs in the peripheral blood of sarcoidosis patients and the correlation between the proportion of Bregs and Tfh cells in the peripheral blood of active sarcoidosis patients were both analyzed by Pearson's correlation test. The findings suggested that the IL-35 level was positively correlated with the proportion of Bregs in the peripheral blood of sarcoidosis patients (*P* = 0.006), but there was no significant correlation between IL-35 level and the proportion of Tfh cells or Tregs. There was no significant correlation between the proportions of Bregs and Tregs in the peripheral blood of active sarcoidosis patients (Supplementary File 2).

#### Significant Increases in p35 and EBI2 mRNA Levels in CD19^+^ Cells of Sarcoidosis Patients

**Figure 5:**
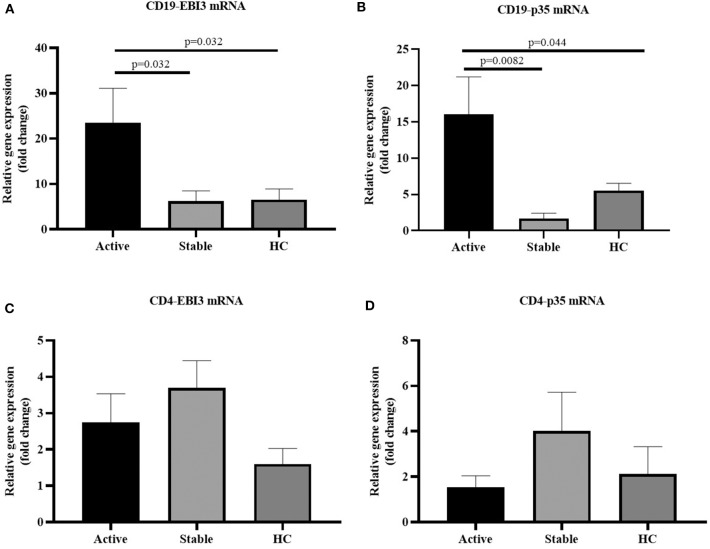
**(A)** The mRNA expression levels of EBI3 in CD19^+^ cells in patients with active and stable sarcoidosis, and healthy controls (HC) (active: *n* = 17, stable: *n* = 18, HC: *n* = 19); **(B)** The mRNA expression levels of p35 in CD19^+^ cells in patients with active and stable sarcoidosis, and healthy controls (HC) (active: *n* = 17, stable: *n* = 18, HC: *n* = 19); **(C)** mRNA expression levels of EBI3 in CD4^+^ cells of patients with active and stable sarcoidosis, and healthy controls (active: *n* = 14, Stable: *n* = 14, HC: *n* = 18); and **(D)** mRNA expression levels of p35 in CD4^+^ cells of patients with active and stable sarcoidosis, and healthy controls (active: *n* = 14, stable: *n* = 14, HC: *n* = 18). The differences between two groups were analyzed by unpaired student's *t*-test.

Peripheral blood mononuclear cells (PBMCs) were extracted from 63 cases with sarcoidosis and 19 healthy controls. Among them, PBMCs were sorted by magnetic beads to harvest CD19^+^ cells from the 35 cases with sarcoidosis (17 cases with active sarcoidosis and 18 cases with stable sarcoidosis) and 19 healthy controls. PBMCs of another 28 cases with sarcoidosis (14 cases with active sarcoidosis and 14 cases with stable sarcoidosis) and 18 healthy controls were sorted by magnetic beads to harvest CD4^+^ cells. RNA was extracted and reverse transcribed into cDNA, then fluorescence-based quantitative real-time PCR was used to detect the expression of the IL-35 subunit (p35, EBI3) and GAPDH as the house-keeping gene. The results showed that the relative gene expression of EBI3 in CD19^+^ B cells of patients with active sarcoidosis was significantly higher as compared to patients with stable sarcoidosis (*P*=0.032) and the control group (Figure 5A, *P* = 0.032). The relative gene expression of p35 in CD19^+^ B cells of patients with active sarcoidosis was significantly higher as compared to patients with stable sarcoidosis (*P* = 0.0082) and the control group (Figure 5B, *P* = 0.044). However, the relative expression of EBI3 and p35 in CD4^+^ T cells was not significantly different compared to patients with stable sarcoidosis or healthy controls (Figure 5C, *P*_1_ = 0.39, *P*_2_ = 0.19; Figure 5D, *P*_1_ = 0.18, *P*_2_ = 0.69).

### The Role of IL-35 in Animal Models

#### There Are Still Sarcoidosis Granulomas in Mice After Intervention With IL-35 Antibody

**Figure 6:**
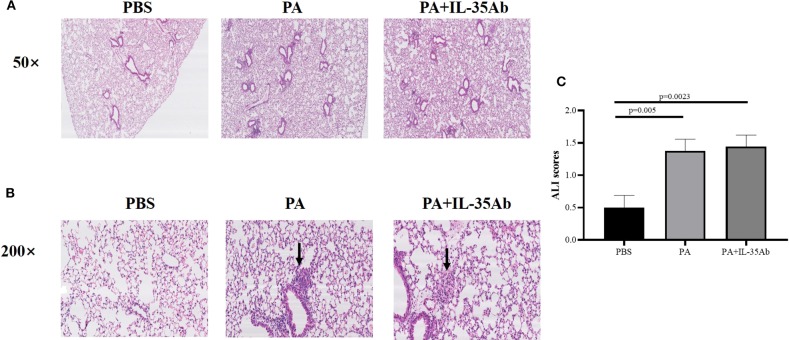
**(A)** Representative images of HE staining of mouse lung tissue specimens in the PBS, PA, and PA+IL-35Ab groups (50×); **(B)** Representative images of HE staining of mouse lung tissue specimens in the PBS, PA, and PA+IL-35Ab groups (200×); and **(C)** Acute lung injury (ALI) scores of pulmonary tissues for mice in the PBS, PA, and PA+IL-35Ab groups (PBS: *n* = 8, PA: *n* = 8, PA+IL-35Ab: *n* = 9). The differences of ALI scores between two groups were analyzed by unpaired student's *t*-test.

In the PA alone and PA+IL-35Ab group, chronic inflammation was found in lung tissues of mice, but there was no significant inflammatory change in the PBS group. The lymphocytes in the PA+IL-35Ab group were increased as compared to the PA group, which showed a trend of aggravated inflammation (Figure 6A).

In the PA alone and the PA+IL-35Ab group, there were loose granulomas in lung tissues of mice, but there was no such change in the PBS group (Figure 6B). In the PA and the PA+IL-35Ab group, we found that the acute injury score of mouse lung tissues was higher as compared to the control group (Figure 6C, *P*_1_ = 0.005, *P*_2_ = 0.0023).

#### IL-35 and IL-21 Levels in BALF of Mice With Sarcoidosis Granulomas Are Increased, While They Are Decreased After Intervention With IL-35 Antibody

**Figure 7:**
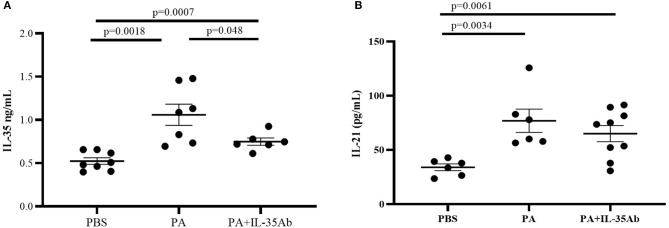
**(A)** IL-35 levels in the BALF of mice in the PBS, PA, and PA+IL-35Ab groups (PBS: *n* = 8, PA: *n* = 7, PA+IL-35Ab: *n* = 6); and **(B)** IL-21 levels in the BALF of mice in the PBS, PA, and PA+IL-35Ab groups (PBS: *n* = 6, PA: *n* = 6, PA+IL-35Ab: *n* = 9). The differences between two groups were analyzed by unpaired student's *t*-test.

Compared to the PBS group, IL-35 levels in the BALF of the PA group (*P* = 0.0007) and the PA+IL-35Ab group (*P* = 0.0018) were significantly elevated, and the IL-35 level in the BALF of the PA+IL-35Ab group was significantly lower than that found in the PA group (Figure 7A, *P* = 0.048). In addition, when compared to the PBS group, IL-21 levels in BALF of the PA group (*P* = 0.0034) and the PA+IL-35Ab group (*P* = 0.0061) were significantly increased. Moreover, the BALF IL-21 level in the PA+IL-35Ab group decreased as compared to the PA group without there being a significant difference (Figure 7B, *P* = 0.37).

#### The Proportions of Bregs in the Peripheral Blood and BALF Increase in Mice With Sarcoidosis Granulomas, While the Proportion of Bregs in the Peripheral Blood Decreases After Intervention With Anti-IL-35 Antibody

**Figure 8:**
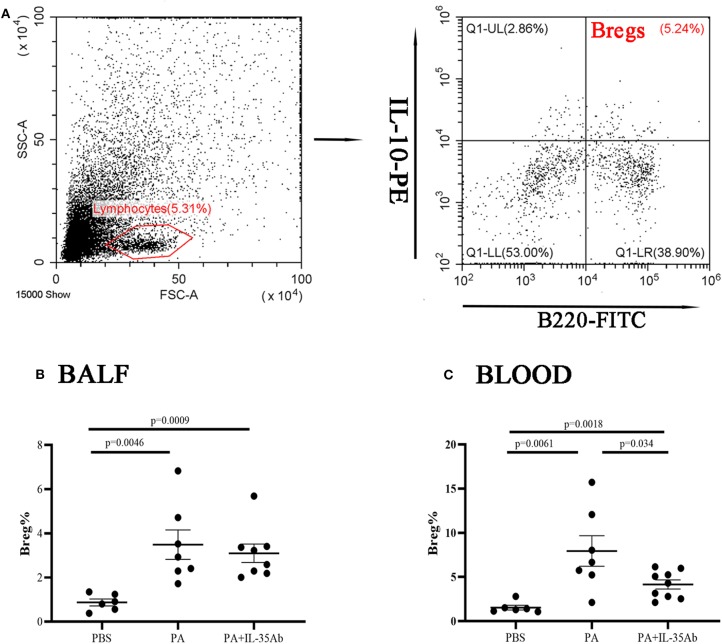
Flow cytometry analysis of Bregs in murine BALF and peripheral blood samples. **(A)** A representative comparative phenotype of B220+IL-10+ cells produced by lymphocytes (Bregs) from a mouse in PA group; **(B)** The proportions of Bregs in the BALF of the PBS, PA, and PA+IL-35Ab groups (PBS: *n* = 6, PA: *n* = 7, PA+IL-35Ab: *n* = 8); and **(C)** The proportions of Bregs in the peripheral blood of the PBS, PA, and PA+IL-35Ab groups (PBS: *n* = 6, PA: *n* = 7, PA+IL-35Ab: *n* = 9). The differences between two groups were analyzed by unpaired student's *t*-test.

In the PA group, the proportion of Bregs in the BALF was significantly higher as compared to the PBS group (*P* = 0.0046), while the proportion of Bregs in the BALF of the PA+IL-35Ab group was lower than that in the PA group without there being any significant difference (*P* = 0.62). In the PA+IL-35Ab group, the proportion of Bregs in the BALF was higher than that found in the PBS group (*P* = 0.0009, Figure 8B). Finally, in the PA group, the proportion of Bregs in the peripheral blood was significantly higher when compared to the PBS group (*P* = 0.0061). In the PA+IL-35Ab group, the proportion of Bregs in the peripheral blood was significantly lower when compared to the PA group (*P* = 0.034), which was higher than that found in the PBS group (*P* = 0.0018, Figure 8C).

#### The Proportions of Tfh Cells in the Peripheral Blood and BALF Increase in Mice With Sarcoidosis Granulomas, and the Proportion of Tfh Cells in the BALF Increases After Intervention With Anti-IL-35 Antibody

**Figure 9:**
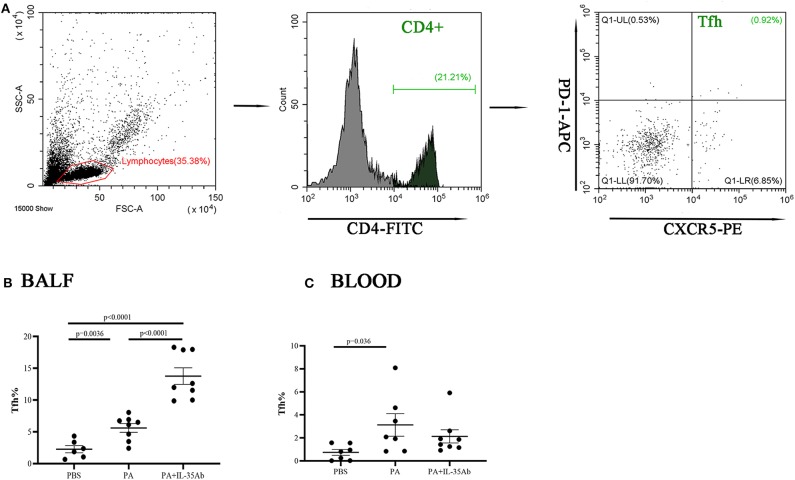
Flow cytometry analysis of Tfh cells in murine BALF and peripheral blood samples. **(A)** A representative comparative phenotype of PD-1+CXCR5+ cells produced by CD4^+^ lymphocytes subsets (Tfh cells) from a mouse in PA group; **(B)** The proportions of Tfh cells in the BALF of the PBS, PA, and PA+IL-35Ab groups (PBS: *n* = 6, PA: *n* = 8, PA+IL-35Ab: *n* = 8); and **(C)** The proportions of Tfh cells in the peripheral blood of the PBS, PA, and PA+IL-35Ab groups (PBS: *n* = 7, PA: *n* = 7, PA+IL-35Ab: *n* = 8). The differences between two groups were analyzed by unpaired student's *t*-test.

In the PA group, the proportion of Tfh cells in the BALF was significantly higher compared to the PBS group (*P* = 0.0036). In the PA+IL-35Ab group, the proportion of Tfh cells in the BALF was significantly higher when compared to the PA group (*P* < 0.0001) and the PBS group (*P* < 0.0001, Figure 9B). The proportion of Tfh cells in the peripheral blood of the PA group was higher when compared to the PBS group (*P* = 0.036); however, there was no significant difference in the proportion of Tfh cells in the peripheral blood after intervention with anti-PA+IL-35 antibody treatment (Figure 9C).

#### The Proportions of Tregs in the Peripheral Blood and BALF Decrease in Mice With Sarcoidosis Granulomas and Increase After Intervention With Anti-IL-35 Antibody

**Figure 10:**
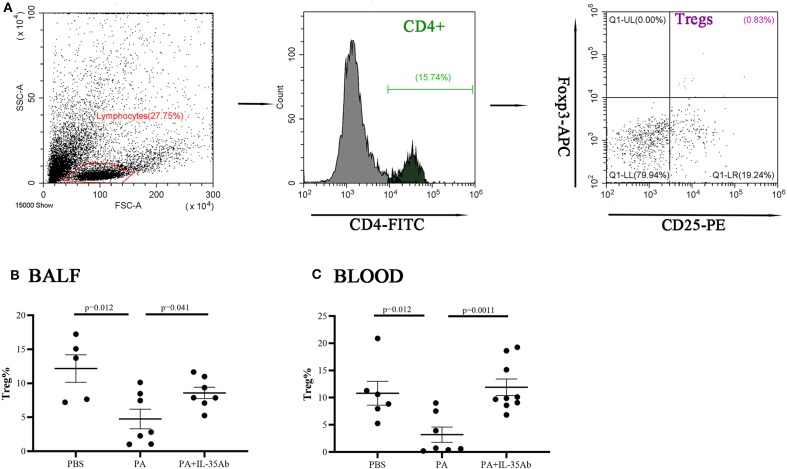
Flow cytometry analysis of Tregs in murine BALF and peripheral blood samples. **(A)** A representative comparative phenotype of CD25^+^Foxp^3+^ cells produced by CD4^+^ lymphocytes subsets (Tregs) from a mouse in PA group; **(B)** The proportions of Tregs in the BALF of the PBS, PA, and PA+IL-35Ab groups (PBS: *n* = 5, PA: *n* = 7, PA+IL-35Ab: *n* = 7); and **(C)** The proportions of Tregs in the peripheral blood of the PBS, PA, and PA+IL-35Ab groups (PBS: *n* = 6, PA: *n* = 7, PA+IL-35Ab: *n* = 9). The differences between two groups were analyzed by unpaired student's *t*-test.

In the PA group, the proportion of Tregs in the BALF was significantly lower when compared to the PBS group (*P* = 0.012). In the PA+IL-35Ab group, the proportion of Tregs in the BALF was significantly higher when compared to the PA group (*P* = 0041; Figure 10B). In addition, in the PA group, the proportion of peripheral blood Tregs was significantly lower when compared to the PBS group (*P* = 0.012), and the proportion of peripheral blood Tregs in the PA+IL-35Ab group was significantly higher than the PA group (*P* = 0.0011, Figure 10C). The detection of Breg+IL-35+ cells and Treg+IL-35+ cells in murine experiments was shown in Supplementary File 3.

## Discussion

IL-35 is secreted by both Tregs and Bregs, and can assist Tregs play important roles in immunosuppression (5, 9, 10). Moreover, IL-35 can induce production of Tregs that produce IL-35 (iTr35), and to promote the proliferation of Tregs, and limit the differentiation and functions of Th17, which is highly significant in maintaining Th17/Treg cell balance (6, 23, 24).

At present, the differential expression of IL-35 has been detected in multiple inflammatory and autoimmune diseases. A study has found that IL-35 controls the development of T cell-dependent colitis, and reduces Th1 and Th17 cytokines in mice, which might evolve into a treatment target for chronic inflammatory bowel disease (11). Bregs play a negative immuno-regulatory effect by secreting IL-10 and IL-35. A previous study showed that CD19^+^ CD24^hi^ CD38^hi^ Bregs inhibit the differentiation of Th1 cells, and do so by expressing IL-10, preventing Th1 cell-mediated immune responses, and assisting Treg differentiation (10).

Currently, the effects of B cell subsets in the pathogenesis of sarcoidosis are only reported in a small number of studies. Van Laar et al. found that there are numerous B cells and IgA-producing plasma cells surrounding the granulomata of the diseased tissues in patients with sarcoidosis, suggesting that B cells may represent a key regulatory factor in sarcoidosis (25). Bouaziz et al. found that transitional blood B cells (CD19^+^ CD24^hi^ CD38^hi^) and IL-10-producing regulatory B cells increased in the peripheral blood of active sarcoidosis patients (26).

The present study found that serum IL-35 level in patients with active sarcoidosis was significantly higher compared to patients with stable sarcoidosis and healthy controls, which indicated that IL-35 level was correlated with the activity of sarcoidosis. In the meantime, it was found that the proportion of Bregs (CD19^+^CD24^+^CD38^+^) in the peripheral blood of patients with active sarcoidosis was significantly higher when compared to patients with stable sarcoidosis and healthy controls. The proportion of Tfh cells (CD4^+^CXCR5^+^PD1^+^) which are closely correlated with the differentiation and maturation of B cells increased significantly in the peripheral blood of patients with active sarcoidosis as compared to patients with stable sarcoidosis and healthy controls. The proportion of Tregs (CD4^+^CD25^+^CD127^−^) in the peripheral blood of patients with active sarcoidosis was significantly lower when compared to patients with stable sarcoidosis and healthy controls.

Pearson's correlation analysis showed that serum IL-35 level was associated with the proportion of Bregs in the peripheral blood, at least to some extent, but was not significantly associated with the proportion of Tregs or Tfh cells. Magnetic bead sorting of CD19^+^ and CD4^+^ cells demonstrated that the relative gene expression of the IL-35 subunits (i.e., p35, and EBI3) in CD19^+^ cells of patients with active sarcoidosis was significantly higher when compared to stable sarcoidosis patients and healthy controls, however the relative gene expression of p35 and EBI3 in CD4^+^ T cells of patients with active sarcoidosis was not significantly different when compared to stable sarcoidosis patients and healthy controls. Collectively, the above evidence suggested that an increase in IL-35 levels in the peripheral blood of patients with active sarcoidosis might predominantly derive from Bregs, and that the presence of IL-35 may play a certain immunoregulatory effect via Tfh cells. However, additional research is needed to further explore why the proportion of Tfh cells in the peripheral blood were not significantly different between patients with active or stable sarcoidosis.

The effects of IL-35 in the pathogenesis of sarcoidosis remain unclear. Significant increases in IL-35 levels for patients with active sarcoidosis may represent a more potent immune dysfunction at this time. Excessive autoimmune responses can be weakened by enhanced immunosuppressive functions, which can partly explain why most patients with sarcoidosis can heal by themselves. The levels of serum IL-35 were dynamically monitored during the follow-up period, and the findings suggested that patients with progressive disease had a decreasing trend, however the IL-35 levels did not change significantly for patients with improved or stable disease conditions, which indicated that decreased levels of serum IL-35 might predict that the disease was severe, although the specific mechanism remains unclear. Thus, from the aspect of an immuno-suppressive effect of IL-35, we speculated that a significant decrease in IL-35 levels accounted for excessively potent autoimmune functions, and aggravation of immune injury, which led to disease progression. However, it would be helpful to have a larger subject group with paired follow up samples for IL-35 analysis.

The mechanism of IL-35 in granulomatous inflammation was further analyzed using a mouse model of sarcoidosis granuloma which was previously established by our study group (20). The findings suggested that IL-35 and IL-21 levels in the BALF of mouse models of sarcoidosis granuloma were elevated, and the proportions of Bregs and Tfh cells in the peripheral blood and BALF also increased, however the proportion of Tregs decreased. These results were consistent with the results of those patients presenting with sarcoidosis, which further suggested that IL-35 levels were increased in granulomatous inflammation. Moreover, IL-35 may predominantly derive from Bregs, and plays a certain immunoregulatory effect via the functional interplay of Tfh cells.

**Figure 11:**
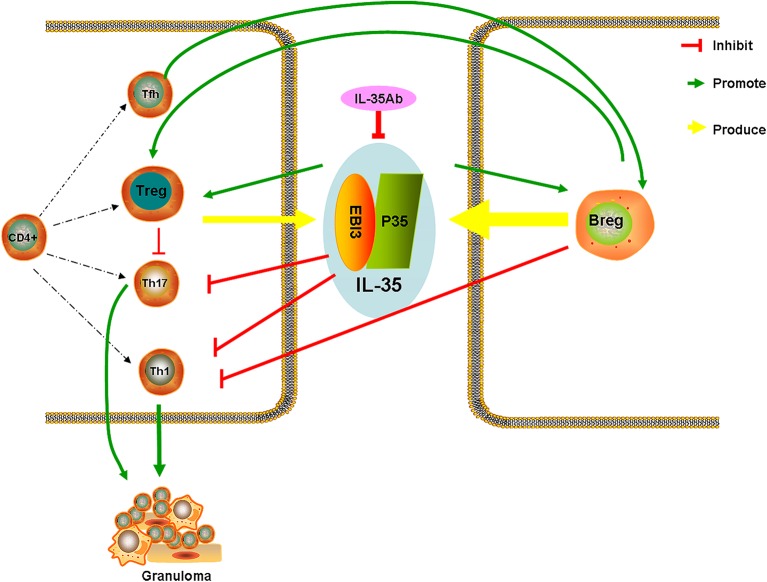
Hypothetical diagram illustrating the mechanisms of IL-35/Breg cells in sarcoidosis.

After intervention with anti-IL-35 antibodies, the pathology indicated that lymphocytes around the granuloma in the PA+IL-35Ab group increased as compared with the PA group, which showed a trend for aggravated inflammation. After intervention with IL-35 antibodies, IL-35 and IL-21 levels were decreased in BALF. Meanwhile, the proportion of Bregs in the peripheral blood declined, the proportion of Tfh cells in BALF increased, and the proportions of Tregs in both the peripheral blood and BALF increased. These results were similar to those obtained from clinical patients with progressive disease, indicating that IL-35 antibodies may weaken the inhibitory effects of IL-35 on granulomatous inflammation, resulting in disease progression. However, the specific mechanism requires further study. Based on the current study results, we proposed a hypothesis whereby increases in Bregs in the peripheral blood of patients with active sarcoidosis secrete more IL-35, thereby having immuno-suppressive effects, which inhibit the immunologic dissonance of Th17/Treg cells in granulomatous inflammation. Tfh cells might also play certain regulatory effects by secreting IL-21 (Figure 11).

## Conclusions

IL-35 levels were significantly elevated in the serum of active sarcoidosis patients. Decreased levels of IL-35 were correlated with aggravation of disease. IL-35 may be used as a potential serological marker for evaluating the activity of sarcoidosis and disease severity. In addition, serum IL-35 levels might be better correlated with Breg cell functions.

## Data Availability Statement

The datasets generated for this study are available on request to the corresponding author.

## Ethics Statement

The study was approved by institutional ethics committee of Shanghai Pulmonary Hospital (No. K17-016). The patients/participants provided their written informed consent to participate in this study.

## Author Contributions

LH, WD, ZM, SJ, DS, and ZYu: experimental design. ZM, SJ, DS, ZYu, ZYi, LQ, WD, and LH: data acquisition and analysis. ZM, SJ, and LH: writing the manuscript. All authors: read and approved the final manuscript.

## Conflict of Interest

The authors declare that the research was conducted in the absence of any commercial or financial relationships that could be construed as a potential conflict of interest.
