# Supplementary material for: Serum IL-35 Levels Are Associated With Activity and Progression of Sarcoidosis
Source: Front Immunol. 2020 May 19;11:977. doi: 10.3389/fimmu.2020.00977 (PMC7248598; doi:10.3389/fimmu.2020.00977)
Supplement: Supplementary file 1 [file Data_Sheet_1.docx]

**Table S1.** **Scoring criteria for acute lung injury (ALI) ^[22]^.**

| **Evaluation features** | | **Scores** | | | | |
| --- | --- | --- | --- | --- | --- | --- |
|  |  | **0** | **1** | **2** | **3** | **4** |
| I | Alveolar congestion | Minimal or little damage | Mild damage | Moderate damage | Severe damage | Maximal damage |
| II | Bleeding | Minimal or little damage | Mild damage | Moderate damage | Severe damage | Maximal damage |
| III | Inflammatory cell infiltration | Minimal or little damage | Mild damage | Moderate damage | Severe damage | Maximal damage |
| IV | Alveolar wall thickening or a hyaline membrane formation | Minimal or little damage | Mild damage | Moderate damage | Severe damage | Maximal damage |

The severity of each feature was scored from 0–4 with 0 representing the least and 4 representing the most severe, and the total score of ALI was the sum of the scores of four features.

**S2. Serum IL-35 levels are positively correlated with the proportions of Bregs for patients with sarcoidosis, but there is no significant correlation with the proportions of Tregs or Tfh cells.**

**
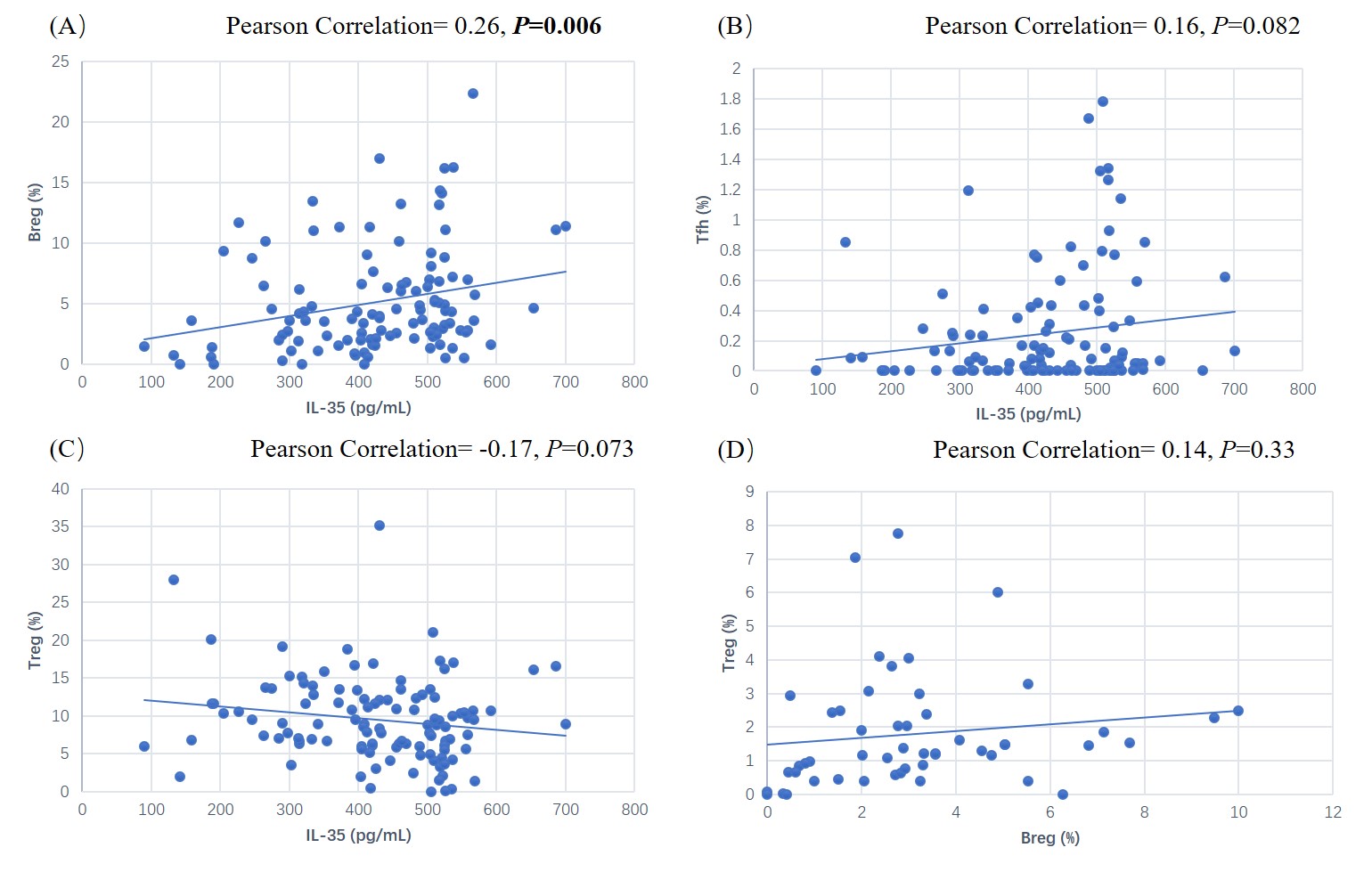
**

**Figure S2** (A) The correlation between IL-35 levels and the proportion of Bregs in the peripheral blood of patients with sarcoidosis (Pearson Correlation= 0.26, n=114, ***P*=0.006)**, (B) The correlation between IL-35 levels and the proportion of Tfh cells in the peripheral blood of patients with sarcoidosis (Pearson Correlation= 0.16, n=114, *P*=0.082), (C) The correlation between IL-35 levels and the proportion of Tregs in the peripheral blood of patients with sarcoidosis (Pearson Correlation= -0.17, n=114, *P*=0.073), and (D) The correlation between the proportion of Bregs and that of Tregs in the peripheral blood of patients with active sarcoidosis (Pearson Correlation= 0.14, n=51, *P*=0.33). The relationship of IL-35 levels with the proportions of Bregs, Tfh cells and Tregs in the peripheral blood of sarcoidosis patients was analyzed by Pearson correlation analysis. The relationship of the proportion of Bregs with that of Tregs in the peripheral blood of active sarcoidosis patients was also analyzed by Pearson correlation analysis.

**S3. IL-35 derives from Breg and Treg cells in murine experiments**

**Methods**

Mice spleens were placed in phosphate-buffered saline (PBS) immediately after sacrifice. After mechanically disruption and centrifugation, cells were isolated, and then processed by erythrocyte lysate. The cells were cultured in RPMI 1640 medium with Cell Stimulation Cocktail (containing Phorbol 12-myristate 13-acetate, lonomycin, Brefeldin A, Monensin, eBioscience, USA) for 16hours, and then the cells were re-suspended in flow cytometry staining buffer solution and divided into two groups. The first group was used for labelling with anti-mouse B220-FITC first, and then the cells were fixed and permeabilized for intracellular staining anti-mouse IL-10-PE, p35-APC and EBI3-PerCP. The second group was labelled by surface staining with anti-mouse CD4-FITC and CD25-PE. and then the cells were fixed and permeabilized with for intracellular staining anti-mouse p35-APC and EBI3-PerCP. Anti-mouse p35-APC and EBI3-PerCP antibodies were purchased from R&D Systems, other antibodies for immunofluorescent staining were purchased from eBioscience. Fluorescence signals were detected in stained cells using flow cytometer (FACSCanto II, BD), and the antibody‐stained cells were analyzed using the Flowjo software. In the first group, Bregs (B220+IL-10+) were gated within the lymphocyte populations in FSC/SSC dot plot, then p35/ EBI3 dot plot was drawn from the Bregs, to gate on IL-35 derived from Bregs (B220+IL-10+p35+EBI3+). In the second group, Tregs (CD4+CD25+) were gated within the lymphocyte populations in FSC/SSC dot plot, then p35/ EBI3 dot plot was drawn from the Tregs, to gate on IL-35 derived from Tregs (CD4+CD25+p35+EBI3+).

**Results**

Breg+IL-35+ cells (Figure S3-1) and Treg+IL-35+ cells (Figure S3-2) could be detected in PBS, PA and PA+IL-35 groups.

**
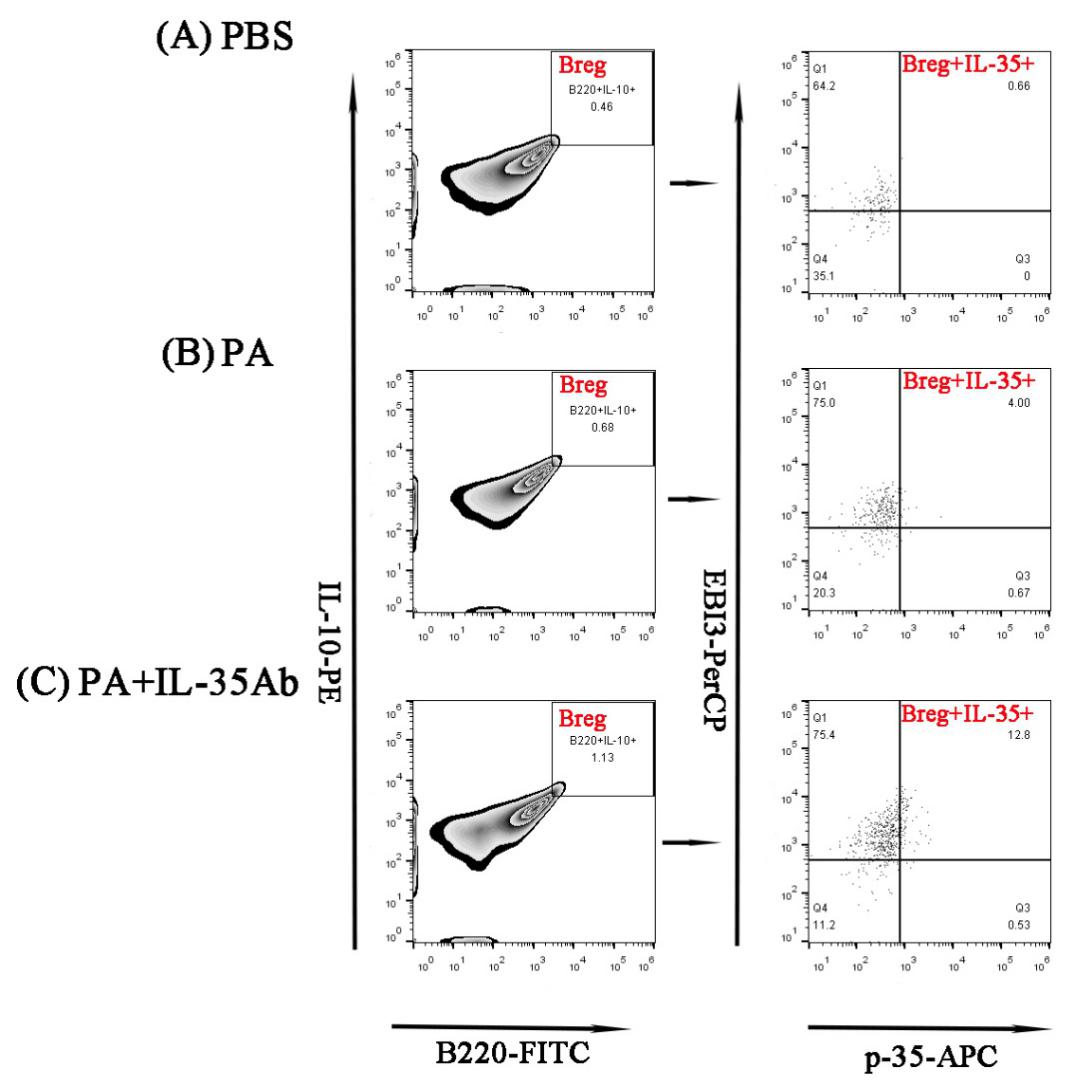
**

**Figure S3-1** (A) A representative comparative phenotype of EBI3+p35+ subsets produced by Bregs (B220+IL-10+) from a mouse in PBS group. (B) A representative comparative phenotype of EBI3+p35+ subsets produced by Bregs (B220+IL-10+) from a mouse in PA group. (C) A representative comparative phenotype of EBI3+p35+ subsets produced by Bregs (B220+IL-10+) from a mouse in PA+IL-35Ab group.


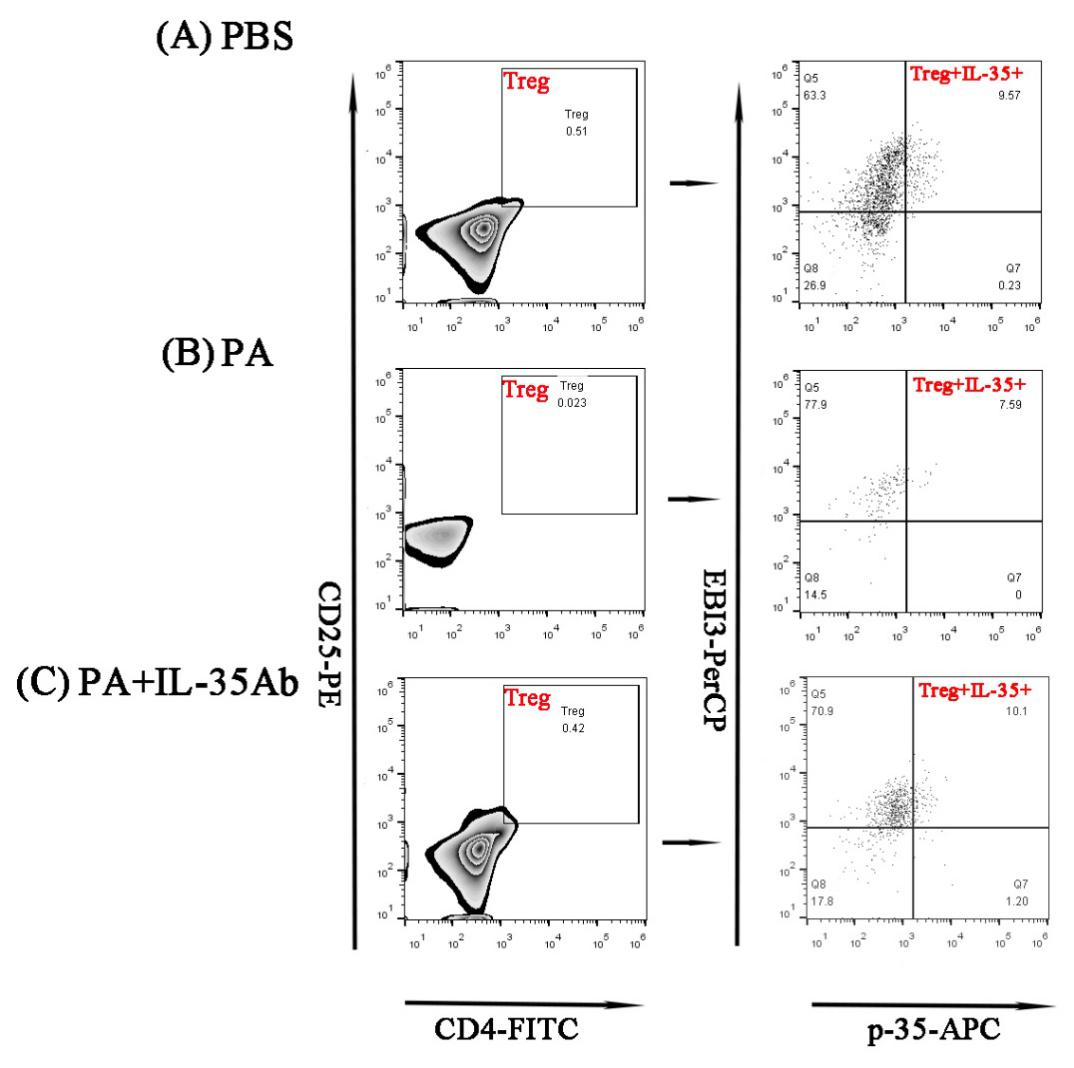


**Figure S3-2** (A) A representative comparative phenotype of EBI3+p35+ subsets produced by Tregs (CD4+CD25+) from a mouse in PBS group. (B) A representative comparative phenotype of EBI3+p35+ subsets produced by Tregs (CD4+CD25+) from a mouse in PA group. (C) A representative comparative phenotype of EBI3+p35+ subsets produced by Tregs (CD4+CD25+) from a mouse in PA+IL-35Ab group.
